# Endemic and Indigenous Diseases

**Published:** 1859-01

**Authors:** Samuel Henry Dickson

**Affiliations:** Prof. of Practice of Medicine in the Jefferson College, Philadelphia


*Endemic and Indigenous Diseases.* By SAMUEL HENRY DICKSON, M. D., Prof. of Practice of Medicine in the Jefferson College, Philadelphia.

Endemicity, a word first used, I believe, by Boudin, in his condite work on Medical Geography and Statistics, is very convenient for the precise expression of a quality apt to be too loosely spoken of by writers. We shall often find the two ideas confounded, of a *local existence,* for an indefinite time, continuous and uninterrupted, and of a *local production,* a truly *indigenous* origin. In Dunglison's great Dictionary. we have suggested, in the definition of the word "indigenous," the exact purport of the word Endemic—the opposite of Exotic. Diseases may be pointed out, perennial in their presence in certain localities, and impossible of extirpation, which were not originally produced there, are therefore not indigenous, and therefore not strictly endemic. Such, for example, are some of the zymotic affections which make their constant appearance on the bills of mortality of large cities—small-pox, scarlatina, pertussis: let us add syphilis and psora to the repulsive list. Others we know belong strictly to the situations where they occur; but we will not be at any difficulty in finding a class of which their local attachment or generation is well known, concerning which, however, we doubt as to the true source. Thus, Plica Polonica is affirmed by many to depend upon the habits of the people subject to it, and not the contingencies of place. The learned author above quoted, gives ague as the striking and apposite illustration of Endemicity; and in truth, all that we clearly know of it, is its generation in, and adhesion to, certain localities. Pellagra is met with in Lombardy, and in the Landes of Southern France; strangers are not attacked with it, nor does it seem to diffuse itself beyond the poorer classes. It is, in its own country, ascribed by most to diet; by some to general modes of living; by a few, to a single article of food; and by some scientific and curious inquirers, to a special vitiation of that single article, maize.

Thus we see, that ague and pellagra, when closely considered, are not to be included in the same category, or definition of Endemicity. A stranger—all strangers are liable in certain places to ague. They are liable under all varieties of circumstance, condition, habits, diet, &c. So it seems to be at Aleppo with the bouton d'Aleppe, to which we might add many others spoken of by authors, such as the Tigretier of Abyssinia, the Astaragaza of Ethiopia. Some act slowly, others rapidly. Goitre and Cretinism may be mentioned here as being no less obscure than malarial ague, and connected strongly, with mere place—Bouton d'Aleppe—universal among natives, but not exclusive; Plica Polenica, exclusive, but not universal; and thus also, of Pellagra, which, so far as appears, attacks natives alone, but not all the natives.

The great characteristic in the natural history of disease, however, which renders them interesting to the general Pathologist, and to the Therapeutist every where, is their varying capacity of dispersion—diffusion—extensive distribution. Some Endemics are circumscribed within narrow limits; some are more or less capable of being distributed; some seem capable of wide diffusion. Every weekly bill of mortality, wherever published, contains a list of deaths from exotic, or truly foreign maladies. The Botanist and Zoologist meet every day with plants and animals, which do not belong to the positions which they now occupy. Lifting my eyes from the page on which I write, I see before me ten varieties of trees, in full foliage, of which only two are indigenous, or of American origin. The fig, the lagurstrimia, the oleander, the varnish tree, the pomegranate, the melia azedarach, are all foreign, most of them, like small pox and cholera, of Eastern origin. Our domestic animals are all imported—the horse, cow, ass, sheep, goat and dog. Every disease must be, or must have been, indigenous somewhere—in that sense, endemic then; if capable of migration, it may extend far away from that central location, and, if the conditions have changed which produced it, we may not find it there now. Even ague seems to have ceased in certain localities, and sometimes appears where unknown previously, and unexpected, owing to the generation or concurrence of the essential conditions. Men take with them animals and vegetables in their migrations; parasites of both kinds follow them against their will. The Norway rat invades all lands. The dandelion, and other foreign weeds, spread themselves abroad unaccountably, and cannot be extirpated. Here, in America, the native man is driven away, or disappears and dies out, and many exotic races have taken his place. Our fields are spread wide with foreign wheat, rye, oats and rice—our orchards planted with fruits of distant origin—the apple, peach, pear, and apricot.

What native diseases have we? what endemics, in the strict and precise sense? We brought with us small pox, and the other exanthemata; we can date the first occurrence of typhus and typhoid: the natives knew nothing of cholera, or yellow fever.

Of Endemicity, in the proper meaning, we have no examples but in the c ass of malarial affections, catalogue them as we may. But we may truly say of any disease, that it is naturalized—when, like our foreign human races, like trees and fruits, and seeds, and weeds, and domestics and parasitic animals, it shows such a congeniality with place, soil, climate, or whatever constitutes the essential characteristics of locality, that it takes firm root, becomes a denizen of the spot—does not die out—refuses to be driven away or exterminated. The favoring contingencies which endow disease with this unhappy tenacity, must vary greatly. Sometimes they may be obvious—as where we reason of the influence of density of population, want of cleanliness, of proper and sufficient food, of ventilation, in keeping up typhus and psora, and syphilis, in the poor of our large cities. Sometimes they are undetected, as when we reason of the presence of typhoid in some of our beautiful Piedmontese situations always subject to it—and of yellow fever in so many of our American towns and cities, where it is either perennial or of regularly annual reproduction, as in Havana and Vera Cruz.

We suppose that we know, historically, when the first yellow fever appeared in Charleston. It is clearly not endemic in the same precise sense in which intermittent and remittent fevers are endemic. It was always treated of by our earlier historians and medical writers—the distinguished Chalmers included—as an exotic, as of foreign origin and introduction. But it may have become here, as we believe it is in many other American localities, naturalized—a denizen of fixed root, ineradicable, and ready to germinate under apposite conditions.

This is a most important question. I, who unequivocally maintain its foreign origin, cannot help feeling a melancholy doubt whether we shall ever be able to exterminate it. I know that Scarlatina is of foreign origin; I am hopeless of its extermination. So of small pox, and measles, and hooping cough, &c.; also, less obviously, of cholera asiatica.

I confess myself occasionally surprised to see so much anxiety shown to establish the indigenous character of this terrible pestilence, which does such fatal injury by its occasional spread among us. I am still more surprised at the inference so promptly drawn by many who believe it thus indigenous, of the inutility of any attempts to interfere with its farther and perpetually renewed introduction from its more certain or endemic seats. Let us consider this matter. Here we have occasional interruptions of its presence or prevalence. We may pass a year, two, three, or four years, without the occurrence of a single case. I suppose that the most ardent controversialist will not deny that the longest of such intervals known, or recorded, have been those in which we have had least intercourse with other places subject to its sway. I believe, though it may not yet be undeniably or certainly proved, that it has never shown itself here, or in our immediate neighborhood, unless when the coincidence occurred of the presence of some vessel within our harbor, or the existence of some cases, or (as Drake tells us in the Mississippi Valley,) of some fomites from its known sources Hence arises, in my mind, a hope—not a very sanguine one, it is true—that being on the extreme limit of the yellow fever zone, we may, by avoiding or preventing the re-introduction of it through its various modes of communication by infected atmosphere in the hold of a ship, or entangled in fomites, succeed in getting rid of it altogether and permanently.

I do not intend to enter upon, or discuss the question of its contagiousness in the familiar sense attached to this word. The poison which gives rise to it, is contained, we all know, in the atmosphere; on board a ship it exists often with such virulence, that all are attacked who venture to remain on board even a short time, not having gained immunity by acclimation, or previous seasoning. Nor does any one doubt that the immediate vicinity of such a ship is dangerous in greater or less degree. And farther, as atmospheric air, with all its intermixtures and qualities, and properties, must be entangled in certain porous articles, it is reasonable, on all fair and theoretic grounds, to believe that many such articles may become fomites, and convey the disease, or the cause of the disease; for we do not, and cannot, know how much may be contained of the poison in any given bulk of air, or how much air must be inhaled or applied to be efficient.

But to return to the general topic of our brief essay. Endemics present each its individual character, so widely separating them in history that they will scarcely bear classification. Diseases that are truly indigenous in any locality, affect natives of that locality chiefly; are, as it were, of elective affinity. Nay, this liability seems sometimes to attach to natives exclusively. Such is the general rule, but there would seem to be at least one remarkable exception:

1. Goitre and Cretinism, Pellagra and Plica Polonica, are endemics which, so far as I am aware, affect only those who are born and continue to reside in the places where they originate.

2. The Bouton d'Aleppe—a cutaneous inflammation peculiar to the region indicated by its name, is universal in its attack on the natives—none of whom say our authorities, escape. But it will occasionally attack visitors also and travellers, whence we infer that it does not require so long time to fix itself upon the constituiion as the former.

3. Malarial endemics fasten specially upon natives, but attack strangers readily. This is true of all the forms of disease ascribed to Malaria.

4. Yellow fever so commonly spoken of, controversially, as endemic and indigenous, is an exception to these general rules above alluded to. Nay—the term exception is too weak to express the value of the fact, if indeed it be properly applicable here. It is far more correct to say that Yellow Fever is in direct contrast to the endemics mentioned, as indeed to all others of which I can find any description. It is so specially apt to attack new comers everywhere, that it is in not a few places called "the stranger's fever." In some localities natives are said to be absolutely exempt; in others, as in Charleston, only comparatively so. The exemption is specially circumscribed—not seeming to be a diffused result of climatic or other general agencies. Thus Semmes says, that the people of the country immediately surrounding Vera Cruz, are more liable to it than strangers from a distance. Our fellow citizens of the parishes surrounding the city we know enjoy not even a comparative exemption. It would appear to be somewhat modified too by contingencies, as thus. The immunity of natives as far as I can learn is complete, wherever Yellow Fever prevails uninterruptedly throughout the whole year—that is unchecked by any winter. Where there is a perfect interval, the disease being unknown in the winter and spring, there is not so complete an exemption of natives. Where, as in Charleston, an interval of a year or more is occasionally enjoyed, natives become subject in greater or less degree—though strangers are notoriously more susceptible of attack. When the disease invades at long intervals, as at Philadelphia, Gibraltar, Cadiz, natives seem to be just as liable as strangers or visitors—at least I am informed of no marked difference.

These diversities in the conduct and history of endemics should lead us to careful and minute inquiry. They probably depend upon differences in the nature and mode of their causation. But the question of cause is always difficult and obscure, and it may be safely asserted that we do not clearly know the origin, the mode of generation, the causative agency productive of any endemic whatever. Read the discussions and observe how illogically and unreasonably the most irrelevant suggestions are pertinaciously urged upon us; how constantly the non-causa pro-causa is argued and vehemently maintained. It has been rendered highly probable that some of this class of maladies depend upon parasitic life—vegetable and animal. Some may be connected with the peculiar character of the soil, of the waters drank, of the food of and the modes of preparing it. But it is hardly too much to affirm that our best knowledge of these matters is, after all, merely conjectural, upon grounds, too, of very equivocal value and relevancy.

Assuming that the facts are as stated, can any one tell us why Trismus is more frequent upon certain islands—the Shetland Isles, those of the Caribbean sea, and our own Long Island, than on any continent? Why Elephantiasis should be met with oftener in Barbadoes than in Ceylon? Hydrocele more frequent in Ceylon than in Barbadoes? Why the Tara should be confined to Siberia, and Beriberi to the East Indies? Why the plague should arise in Egypt and not in Cuba; and the yellow fever in Havana and not in Cairo.

We are more familiar with the endemic presence of malarial fevers than any other; and we have speculated most abundantly upon their source. We are satisfied that we know the reason why typhus prevails in Dublin and St. Giles and on board an emigrant vessel or a slave ship. Perhaps it is not quite so clear why its congener typhoid or enteric fever, attacks some of our most beautiful country villages, and salubrious summer resorts. Every young possessor of a diploma can tell you when he meets with cholera, from what foul drain or noisome sewer its generating effluvium, has escaped in the neighborhood. How definitely are we instructed along our Southern coast in the doctrine of causation of the hæmagastric pestilence which casts its dark shadow over our hopes and prospects; and desolates our streets and houses! Nowhere is the non-causa pro-causa so arrogantly and polemically thrust upon us as in this difficult and interesting investigation. In one locality the dreaded malady was dug up with newly turned earth, where gas-pipes were laid or drains cut. In another, it was the product of a foul fermenting cess-pool; in another, of a slaughter-pen. In the dense lanes of a crowded city it arose from the contingencies to which typhus is ascribed generally; and in the scattered and rural residences of a sandy seashore village, from those which give out malaria or produce intermittents, none being then or there present.

To pronounce any malady of specific character to be indigenous, when it only occasionally presents itself in the locality indicated, might be said in the very outset to be illogical. Its relation is not absolutely to place, if it be not always found in that place. To what other contingency then, is it absolutely related? The inquiry has not yet been satisfactorily answered—the causa sine qua non, is yet a matter in dispute. Not heat, nor filth, vegetable nor animal, nor moisture, nor dryness; for these have all varied separately and in combination, without affecting the results. It has been seen where the surface was unbroken—absent when it was widely turned up. It has failed to show itself for consecutive years of great variety of condition. It has shown itself in consecutive years of equally marked variety. Last year, cold and dry as was the summer, it assailed a pleasant and neat village in the vicinity of Charleston, nor did the city altogether escape. This season, while hot and dry, it invaded the city first, and has spared its neighbors. They are turning up no soil, laying no gas-pipes, digging no drains. The streets are extensively paved, and the police are not accused of neglecting them or failing to keep them clean. Yet there, we have the same intruder which forty years ago was ascribed to unpaved streets, undrained surfaces, and neglected scavenger's observances. It is plain that we have failed in this direction to indicate the cause—or by efforts suggested by these ancient views to weaken its force or arrest its effects. What may be true elsewhere I know not. Of that locality however, I think, that we may affirm that on no single occasion within the period indicated has yellow fever existed unpreceded by two contingencies. 1. The previous existence of the pestilence in some city or cities with which it was in commercial intercourse; and 2. The presence of some vessel from that or those places in its port.

I said that I was oppressed by a painful doubt whether my native city, is not "within the yellow fever zone," in a certain sense; that is, whether its climate is not so congenial with the detested cause of that terrible plague as to afford it a fostering nidus, and to preserve it unextinguished from year to year. The winter of Philadelphia is certainly fatal to it. Yellow fever cannot lie over from year to year in that locality. Nor can it in Norfolk, or even in Wilmington. It seems to me altogether futile to contend for its spontaneous production as an endemic in either of those localities, as indigenous or not clearly exotic. But advancing Southward the question assumes a serious aspect. If in Havana and Vera Cruz it be perennial—waking into annual vigour like flower and fruit—where shall we draw the line? The orange and lime live doubtfully on the coast of South-Carolina; why not the Yellow fever germ—or if I must not be allowed the phrase—the poison producing yellow fever, why not also persistent there? I hope it is not. The chief ground of that hope is the uniform influence of all long continued interruptions of commerce upon this point. Our history shows that both our wars and our embargo and nonintercourse act kept us clear of our dreaded guest. And since my attention has been drawn to the subject, I am not aware that any single case has occured in Charleston when there has not been an infected vessel, either at the time, or recently, previously in its harbor. I am not at all embarrassed with difficulties of transmission, or the impossibility of tracing the imported cause from point to point, or the rapidity of its efficient diffusion when imported. The ingenuity of the smuggler, the eccentric movements of the wild reveller, the daring of the reluctant prisoner, afford me analogies enough of ready explanation. Add to these the observed rapidity of spread of the most palpable contagions, and the total impossibility of limiting or tracing their modes of communication, and there is nothing to embarrass the formation of an opinion as to the necessary connection between the ships lying at quarrantine in the harbor, and the cases occurring in and all over the city.

No satisfactory solution of this problem will be arrived at until we institute for a few successive years an absolute and entire interruption of intercourse during the summer months with the ever-infected places where the hæmagastric pestilence, though not indigenous, nor originally a native, has yet become a denizen, ineradicable, and always active and self-dispensing. We have here, it seems, the materials always ready for explosion; let us keep out the spark, whether it be brought in the form of a vegetable sporule, an animal ovum, an organic or inorganic substance, a living parasite of either kingdom, a mere poisonous bulk of infected atmosphere, or an atom of contagion. What matters this dispute about words! Let us examine if the approach, the importation of some materies morbi, palpable or impalpable, is an essential point; if the coincidence is uniform; if the visit or presence of a *vessel* from an infected place be necessary.

Next, if having kept away this mode of infection, we can trace to *persons* or *fomites* the introduction of the disease—this also seems not unfairly deduced from known facts—and lastly, if we can ascertain that around the body of the sick there is diffused an efficient poison capable of generating the disease in a healthy body. We shall then have taken it out of the category of emdemics, as having no exclusive relation to place any more than small pox or measles, which are now spoken of no longer as indigenous anywhere, or endemic in or of any special locality.—*Charleston Medical Journal.*

